# Gastric Antral Vascular Ectasia (GAVE), or “Watermelon Stomach,” Is Associated With Chronic Occult Gastrointestinal Blood Loss, Leading to Iron-Deficiency Anaemia and an Increased Risk of Falls Due to Fatigue and Reduced Physiological Reserve

**DOI:** 10.7759/cureus.110574

**Published:** 2026-06-10

**Authors:** Hussein Abu Rabia

**Affiliations:** 1 Medicine and Surgery, NHS, Manchester, GBR

**Keywords:** collapse, frailty syndrome, gastric antral vascular ectasia (gave), iron deficiency anaemia (ida). anaemia of chronic disease(acd), watermelon stomach

## Abstract

Gastric antral vascular ectasia (GAVE) is an under-recognised cause of chronic gastrointestinal blood loss and iron-deficiency anaemia, caused by dilated antral vessels that cause chronic gastrointestinal bleeding, gradually depleting iron stores and leading to reduced haemoglobin levels. Its presentation is often subtle and may occur without overt gastrointestinal bleeding. We report an 82-year-old patient admitted following an unwitnessed fall, associated with postural hypotension and normocytic anaemia (Hb 90 g/L). Oesophagogastroduodenoscopy (OGD) demonstrated linear GAVE in the gastric antrum without active bleeding. The patient also sustained a fragility pelvic fracture.

Intravenous iron therapy was administered to correct severe iron deficiency, resulting in improved haemoglobin levels and resolution of underlying clinical symptoms. This case highlights an important and often under-recognised presentation of GAVE, in which anaemia manifests predominantly through falls or orthostatic features rather than gastrointestinal symptoms. Early recognition of this pattern is crucial, as targeted treatment may reduce the risk of recurrent falls, fractures, and subsequent functional decline in older adults.

## Introduction

Falls are among the most common and serious geriatric syndromes, frequently leading to loss of independence, institutionalisation, and long-term disability. They are often multifactorial in origin, with systemic contributors such as orthostatic hypotension playing a central role. A fall may occur when a postural drop in blood pressure leads to cerebral hypoperfusion, resulting in dizziness, presyncope, or syncope. Despite increasing clinical focus, fall prevention remains challenging, particularly in older adults with multimorbidity and polypharmacy [1,2].

Anaemia is an important and often under-recognised contributor to falls. It can exacerbate fatigue, reduce exercise tolerance, impair cerebral oxygen delivery, and increase postural instability. In older adults, anaemia is frequently chronic and insidious, with non-specific or absent symptoms, meaning the underlying cause may remain undetected without systematic evaluation [3].

Gastric antral vascular ectasia (GAVE), also known as “watermelon stomach,” is an uncommon but clinically important cause of chronic gastrointestinal blood loss. It accounts for approximately 4% of non-variceal upper gastrointestinal bleeding in older adults and is commonly associated with chronic iron-deficiency or normocytic anaemia [4]. Diagnosis is established endoscopically, typically demonstrating characteristic longitudinal erythematous streaks or diffuse vascular changes in the gastric antrum.

GAVE may present without overt gastrointestinal bleeding, making diagnosis challenging and often delayed. Its recognition is crucial, as persistent anaemia contributes significantly to frailty, reduced physiological reserve, increased fall risk, and functional decline in older adults. This case describes a GAVE identified during inpatient investigation following a fall.

## Case presentation

An 82-year-old woman presented to the emergency department after an unwitnessed fall at home, followed by pelvic pain and reduced mobility. She reported no preceding dizziness, chest pain, palpitations, or loss of consciousness, and there was no head injury. Examination demonstrated suprapubic tenderness and tenderness over the left inferior pubic ramus. Pelvic radiography confirmed a left inferior pubic ramus fracture and a suspected chronic right sacral alar fracture.

Initial blood tests confirmed normocytic anaemia, with haemoglobin 90 g/L (reference range 115-165 g/L) despite a normal mean corpuscular volume (MCV) and haematinic profile (Table 1). This accentuates that iron deficiency anaemia may present with preserved indices and highlights the need for further evaluation (e.g., iron studies) when clinical suspicion persists, to avoid delayed diagnosis and treatment. There was no clinical evidence of overt gastrointestinal bleeding, and the haematology review found no convincing evidence of haemolysis. Further laboratory evaluation, including ferritin, confirmed iron deficiency, while vitamin B12 and folate levels were within normal limits.

**Table 1:** Comparative Laboratory Results at Admission and Discharge

Investigation	Results on admission	Results on discharge	Reference Range
Haemoglobin	90g/L	105g/L	115-165 g/L
Haematocrit	0.292 L/LO	0.325L/LO	L0.370-0.470 L/L
Mean corpuscular volume	95.7fL	94.90 fL	80-98 fL
Serum iron	3.0	Not repeated	5.8-34.5 µmol/L
Transferrin saturation4	4.6%	Not repeated	15-45%
Urea	5.6	6.5	2.5 – 7.8 mmol/L
B12	221ng/L	Not repeated	197 - 771 ng/L
Folate	8.3	Not repeated	3.9 – 20.0 ug/L

Lying and standing blood pressure measurements demonstrated symptomatic orthostatic hypotension, with systolic blood pressure falling from 136 mmHg while supine to 105 mmHg after one minute of standing. She was commenced on fludrocortisone 100 micrograms once daily for symptomatic orthostatic hypotension in the context of hypovolaemia, to improve intravascular volume and reduce orthostatic symptoms.

Her past medical history included chronic iron-deficiency anaemia, atrial fibrillation treated with oral anticoagulation, hypertension, orthostatic hypotension, and breast cancer treated with lumpectomy. She had undergone a normal colonoscopy 18 months before admission, although a recent faecal immunochemical test was positive.

**Figure 1:**
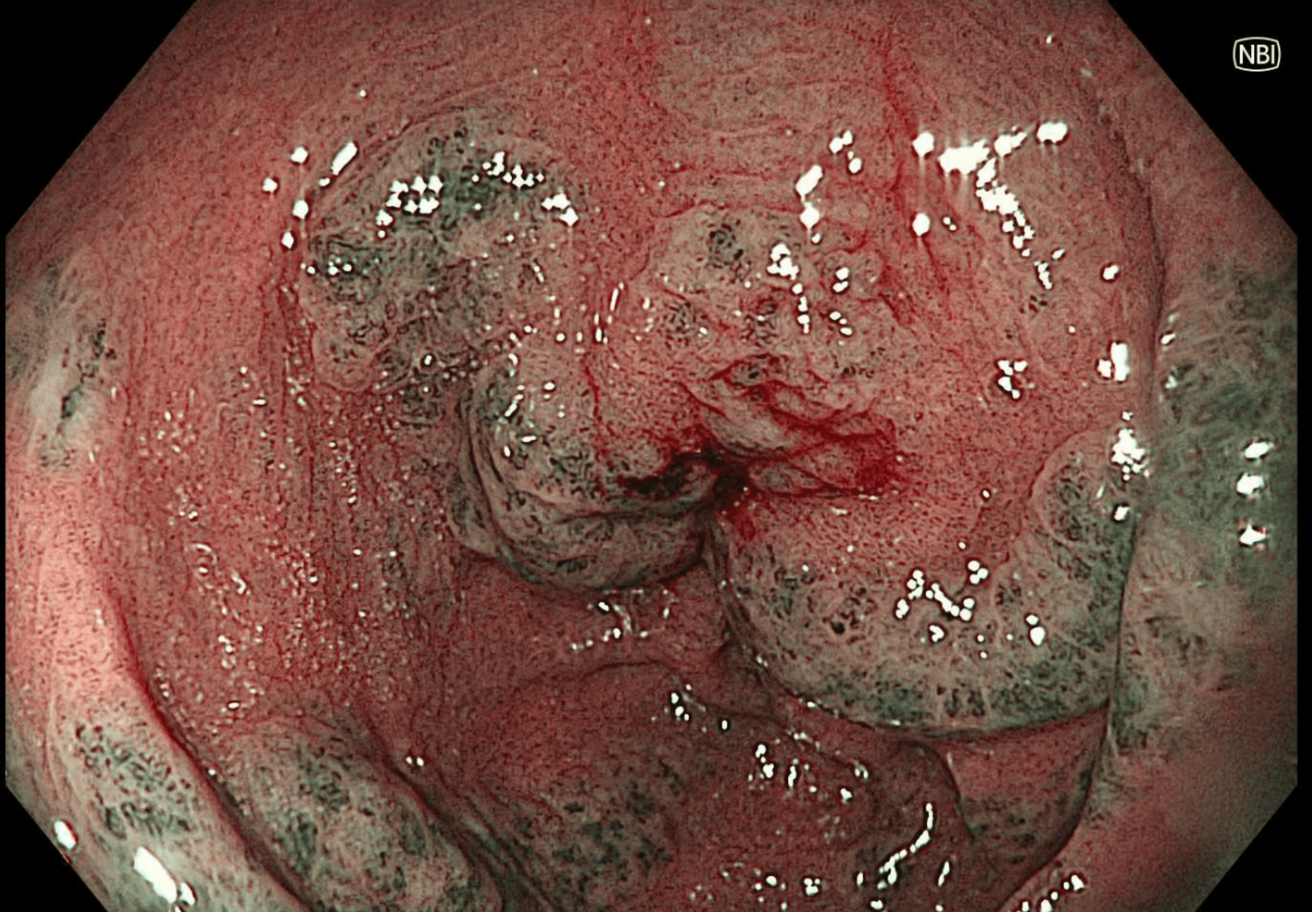
Gastric Antral Vascular Ectasia (GAVE)

Following admission to the geriatric medicine ward for a comprehensive geriatric assessment, she was found to be hypovolaemic and was treated with intravenous fluids. Oesophagogastroduodenoscopy (OGD) demonstrated linear GAVE in the antrum, without active bleeding (Figure 1). The endoscopic findings were considered the likely cause of her chronic iron-deficiency anaemia. She received intravenous iron replacement. Haemoglobin improved gradually following intravenous iron therapy, increasing from 90 g/L on admission to [insert value] g/L before discharge over [insert timeframe] (Table 1). This is consistent with an early treatment response; however, a more substantial rise would be anticipated over subsequent weeks, reflecting the lifespan of erythrocytes and the time required for effective erythropoiesis. Outpatient argon plasma coagulation (APC) and ongoing haematological monitoring were arranged to manage GAVE-related chronic blood loss and reduce recurrence of iron-deficiency anaemia, despite the absence of active bleeding at endoscopy. This case describes gastric antral vascular ectasia presenting as chronic iron-deficiency anaemia in an older patient admitted following a fall. In the absence of other identifiable causes and with supportive iron-deficiency indices, the GAVE identified on endoscopy was considered the plausible source of chronic blood loss rather than an incidental finding.

Pelvic imaging following the fall showed a left inferior pubic ramus fracture as well as a suspected chronic right sacral alar fracture. A fracture risk assessment tool (FRAX) assessment estimated her risk of a major osteoporotic fracture at 45% and hip fracture at 33%, prompting treatment with IV zoledronate.

**Figure 2:**
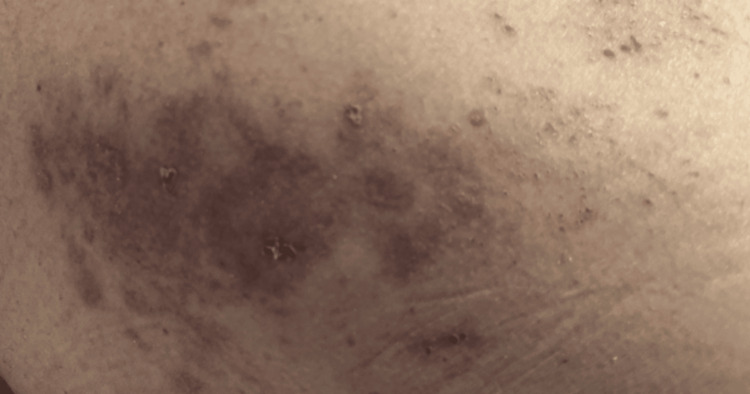
Postherpetic Hyperpigmentation

During her admission to the old person laising and assessment ward, she developed a painful, erythematous rash over the L4-5 dermatome, diagnosed clinically as shingles (Figure 2). 

The patient was discharged with a short course of opioid analgesia to manage fracture-related pain and postherpetic neuralgia, and was encouraged to mobilise as she was able. Her postural hypotension settled without the need for medication, allowing fludrocortisone to be discontinued. She has been arranged for a follow-up endoscopy with planned argon plasma coagulation, and will receive intravenous iron infusions on an annual basis if clinically required.

## Discussion

This case highlights a common clinical challenge in geriatric medicine: Chronic iron-deficiency anaemia in an older patient with multiple comorbidities, orthostatic hypotension, and recurrent falls. In this patient, the underlying cause of the anaemia was GAVE, identified on OGD performed during the admission.

GAVE is characterised endoscopically by linear erythematous vascular streaks in the gastric antrum, often referred to as the classic “watermelon stomach” appearance (Figure 2). Although some patients present with overt gastrointestinal bleeding, many develop chronic occult blood loss resulting in iron-deficiency anaemia. In early disease, the mean corpuscular volume may remain within the normal range despite significant iron depletion [5,6].

The exact pathophysiology of GAVE is not fully understood. Proposed mechanisms include abnormal antral motility causing repetitive mucosal trauma, neurohormonal dysregulation, and associations with autoimmune and connective tissue disorders. Histologically, this results in dilated mucosal capillaries, fibrin thrombi, and vascular congestion [7,8].

Management is determined by the severity of anaemia, evidence of ongoing blood loss, and the patient’s overall clinical condition. In the absence of overt bleeding, initial treatment consists of iron replacement and correction of any associated hypovolaemia. Intravenous iron is often preferred in older adults, as it replenishes iron stores more rapidly, avoids gastrointestinal adverse effects, and reduces the burden of polypharmacy. Red blood cell transfusion is generally reserved for patients with symptomatic anaemia, significant cardiovascular comorbidity, or haemodynamic instability [9].

Endoscopic therapy is the mainstay for recurrent bleeding or iron-deficiency anaemia that persists despite adequate iron replacement. APC is the most widely used first-line modality due to its accessibility and efficacy in achieving haemostasis. Alternative endoscopic approaches include endoscopic band ligation (EBL) and radiofrequency ablation (RFA), both of which may be considered in recurrent or refractory disease. Medical therapies have been reported in selected cases, but the evidence base remains limited and inconsistent. Surgical intervention, typically distal gastrectomy, is rarely required and is reserved for cases that fail all endoscopic and medical management strategies [6,7,10].

In this patient, endoscopy showed no evidence of active or recent bleeding, and haemoglobin stabilised following intravenous iron therapy. A conservative management strategy was therefore appropriate, with planned outpatient APC and ongoing haemoglobin surveillance.

The fall was likely multifactorial. Symptomatic orthostatic hypotension was confirmed on postural blood pressure measurements, while chronic anaemia may have further contributed through reduced exercise tolerance, presyncope, and diminished physiological reserve. The combination of these factors significantly increased her risk of falls and subsequent fragility injury.

Comprehensive geriatric assessment was central to her management, providing a structured, multidisciplinary approach to the fracture, orthostatic hypotension, chronic anaemia, and associated functional decline.

This case underscores the importance of considering GAVE in older adults with unexplained iron-deficiency anaemia, particularly when prior lower gastrointestinal investigations are unremarkable. Recognition of the underlying diagnosis enables targeted endoscopic therapy and optimised medical management, with the potential to reduce recurrence of anaemia, mitigate fall risk, and preserve functional status.

## Conclusions

Most falls in older adults are multifactorial, often driven by a combination of intrinsic and extrinsic factors; the exact precipitant can be difficult to identify in a busy environment. Comprehensive geriatric assessment (CGA) is essential for all patients presenting with falls to systematically identify and address the contributory underlying cause or causes. GAVE should be considered in older adults with recurrent or unexplained iron-deficiency anaemia, particularly when prior gastrointestinal investigations are unrevealing. Early diagnosis via OGD enables targeted therapy and may prevent complications such as recurrent anaemia, functional decline, and falls. Ongoing multidisciplinary follow-up is often required to reduce recurrence and optimise outcomes. Orthostatic hypotension is common in older patients and should prompt careful evaluation for underlying causes, including hypovolaemia, anaemia, autonomic dysfunction, and medication effects, before initiating or escalating pharmacological therapy.

In this case, the recurrent falls were likely multifactorial, with postural hypotension secondary to chronic anaemia and reduced effective circulating volume as key contributors. Identification of GAVE as the underlying driver of anaemia allows targeted endoscopic treatment and may reduce future falls, fragility fractures, and the need for long-term pharmacological management of orthostatic hypotension.
